# Post-polymerisation approaches for the rapid modification of conjugated polymer properties

**DOI:** 10.1039/d2mh00519k

**Published:** 2022-08-11

**Authors:** Martina Rimmele, Florian Glöcklhofer, Martin Heeney

**Affiliations:** Department of Chemistry and Centre for Processable Electronics, Imperial College London London W12 0BZ UK f.glocklhofer@imperial.ac.uk; KAUST Solar Center, Division of Physical Sciences and Engineering, King Abdullah University of Science and Technology Thuwal 23955-6900 Saudi Arabia martin.heeney@kaust.edu.sa

## Abstract

Post-polymerisation functionalisation provides a facile and efficient way for the introduction of functional groups on the backbone of conjugated polymers. Using post-polymerisation functionalisation approaches, the polymer chain length is usually not affected, meaning that the resulting polymers only differ in their attached functional groups or side chains, which makes them particularly interesting for investigating the influence of the different groups on the polymer properties. For such functionalisations, highly efficient and selective reactions are needed to avoid the formation of complex mixtures or permanent defects in the polymer backbone. A variety of suitable synthetic approaches and reactions that fulfil these criteria have been identified and reported. In this review, a thorough overview is given of the post-polymerisation functionalisations reported to date, with the methods grouped based on the type of reaction used: cycloaddition, oxidation/reduction, nucleophilic aromatic substitution, or halogenation and subsequent cross-coupling reaction. Instead of modifications on the aliphatic side chains of the conjugated polymers, we focus on modifications directly on the conjugated backbones, as these have the most pronounced effect on the optical and electronic properties. Some of the discussed materials have been used in applications, ranging from solar cells to bioelectronics. By providing an overview of this versatile and expanding field for the first time, we showcase post-polymerisation functionalisation as an exciting pathway for the creation of new conjugated materials for a range of applications.

## Abstract 2

We summarise chemical approaches to modify the properties of conjugated polymers by post-polymerisation functionalisation directly on the backbone.

## Graphical abstract

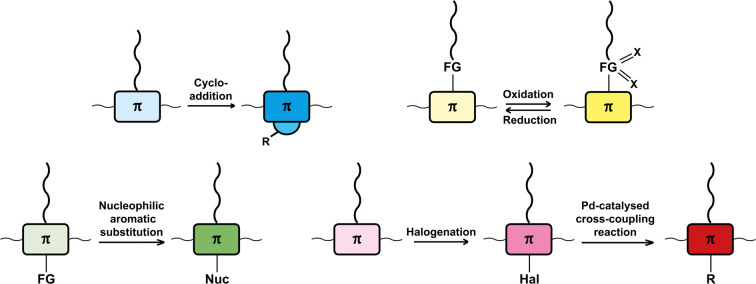

## Introduction

The remarkable optical and electronic properties of conjugated polymers (CPs), combined with their good processability and mechanical flexibility, has prompted much interest in their use for a variety of applications, ranging from energy storage to optoelectronic devices.^1,2^ Among the most important applications are organic photovoltaics and photodetectors (OPVs and OPDs), organic light-emitting diodes (OLEDs), sensors, and organic field-effect transistors (OFETs).^3–6^ The utilisation of CPs can enable new device properties, such as mechanical flexibility and optical transparency, as well as facilitate large-scale fabrication *via* potentially lower-cost printing based approaches.^7–10^

**Fig. 1:**
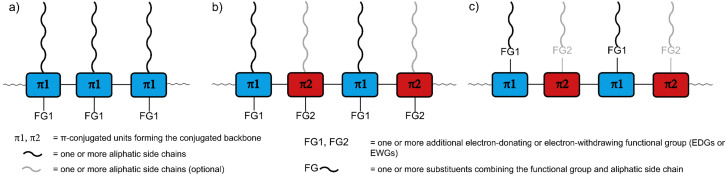
Schematic representation of conjugated polymers with (a) a single type of π-conjugated unit (π1) forming the conjugated backbone as well as aliphatic side chains and (optional) additional functional groups (FG1) as the other two parts of the conjugated polymer, (b) alternating donor and acceptor units (π1 and π2) forming the conjugated backbone as well as aliphatic side chains and (optional) additional functional groups, which are not necessarily present in both units, and (c) aliphatic side chains and additional functional groups combined into the same substituents.

For all CP applications, a number of molecular design features need to be considered. Importantly, CPs usually consist of the following parts (Fig. 1a–c): (1) the π-conjugated backbone that determines most of the optical and electronic properties, (2) aliphatic side chains that influence the solid-state packing and render the polymers soluble, crucial for processing the polymers from solution, and – optionally – (3) additional functional groups for fine-tuning of the optical and electronic properties, either electron-withdrawing groups (EWGs) or electron-donating groups (EDGs) with stronger electron-donating properties than the aliphatic side chains.^11–13^ The side chains and the additional functional groups are often combined into the same substituents attached to the π-conjugated backbone (Fig. 1c). Each of the three parts needs to be carefully selected, as they determine the properties of the polymers, most importantly the frontier molecular orbital (HOMO and LUMO) energy levels and, consequently, the band gap. The band gap is important as it determines the wavelength of light emitted in OLED applications but also the fraction of the solar spectrum that is absorbed in OPV/OPD applications. However, in addition to band gap requirements, the frontier molecular orbital energy levels of conjugated polymers also need to be carefully controlled in order to provide suitable matching with the work function of the electrodes or to facilitate desired charge-separation or -transfer processes between component materials.^14^

Besides the HOMO and LUMO energy levels and band gap, other properties such as good film-forming ability and high charge carrier mobility need to be considered in the polymer design.^15,16^ By thoughtfully selecting the three parts of CPs and designing the monomers accordingly, all these properties can be tuned and controlled. For a narrow band gap, the HOMO energy level needs to be raised or the LUMO energy level lowered (or both). Functional groups can be attached to the monomers to achieve this effect, making use of mesomeric and inductive effects to modify the electronic character: The attachment of EDGs can increase the HOMO and LUMO energy level of the polymer, while the attachment of EWGs can decrease the energy levels. In a different approach, co-polymerisation of electron-rich donor (D) and electron-deficient acceptor (A) monomers can be used to obtain alternating D–A polymers (Fig. 1b and c).^15,17,18^ In such D–A polymers, the bandgap can be significantly narrowed as a result of the hybridisation of the energy levels of the donor and acceptor units. D–A interactions are often proposed to lead to increased double bond character between the D and A units and therefore to increased backbone planarity, but we note that in a recently reported crystal structure of a D–A dimer such effects could not be observed.^19^ Regarding the polymer design, it is noteworthy that the electron-rich or -deficient character of the D and A units can be an inherent property of their conjugated system, but EDGs or EWGs can be attached to further strengthen this character and shift the energy levels.^20–24^

Another important aspect to consider in the polymer design is the self-assembly of the polymers, as the backbone orientation and packing structure can have a significant impact on the charge carrier mobility and, hence, on the device performance.^25,26^ The choice of side chains, which are needed to render the polymers soluble, has a particularly strong impact here. The side chains lead to spatial separation of the backbones, diminishing the molecular orbital overlap and, therefore, limiting the hopping of charges between polymer chains. While bulky side chains can additionally lead to twisting of the polymer backbone, preventing the formation of ordered domains, linear side chains can enable interdigitation and, consequently, closer packing and reduced spatial separation of the backbones, leading to better charge carrier mobility.^27^ Moreover, the formation of ordered domains can also be enhanced by regioregularity, which generally has a positive effect on the charge carrier mobilities.^25,28,29^ On the other hand, increased spatial separation of the backbones can be favourable in OLED devices, where close packing can lead to excimer formation, one of the processes that can result in quenching of the excited state in the solid state.

Traditionally, both the side chains and the additional functional groups (for tuning the self-assembly and the optical and electronic properties, respectively) are introduced in the monomers. These monomers are then (co-)polymerised using a broad variety of techniques that enable the formation of carbon–carbon single bonds. However, the synthesis of these functionalised monomers is often laborious, which inhibits facile changes to the attached groups. If researchers wish to attach a different side chain or functional group to the polymer, they usually need to start again early in the synthesis sequence, namely with the synthesis of the monomer(s).^21,30,31^ In addition, the range of functional groups that can be introduced *via* the available polymerisation techniques is limited; some functional groups cause side reactions, lead to a loss of control over the molecular weight, or completely inhibit the polymerisation reaction.

Post-polymerisation functionalisation can provide a solution to this problem. The approach dates back to Hermann Staudinger, the 1953 Nobel laureate in chemistry, who reported what he called “polymer analogous reactions”, describing them as reactions that lead to polymer derivatives with similar molecular weight as the starting polymers.^32–35^ The idea is that polymers that contain certain functional groups or attachment points on the repeat units are available for functionalisation in a consecutive step. Such functionalisation after the polymerisation step, nowadays known as post-polymerisation functionalisation or modification, offers several advantages, including facilitated introduction of a range of different functional groups, which enables the generation of large sets of polymers with less synthetic effort. Importantly, the polymer chain length is usually not affected by these functionalisations, making it a very powerful approach for creating libraries of comparable functionalised polymers.

It should be noted that reactions suitable for post-polymerisation functionalisation need to be very efficient and high yielding, with side reactions not being tolerated, as side reactions that occur on the polymer lead to defects that cannot be corrected by removal of the side product. Furthermore, high reactivity is crucial, as the accessibility of the polymer backbones is lowered by the sterically demanding nature of the polymers, and low reactivity can lead to complex mixtures of polymers with varying degrees of functionalisation.^32^ Today, very efficient transformations are available that can be used for post-polymerisation functionalisation, but so far, most of the effort has focussed on post-polymerisation functionalisation of non-conjugated polymers or on the side chains of conjugated polymers.^32,36–46^ Unfortunately, the optical and electronic properties of conjugated polymers are usually not significantly influenced by reactions on the side chains, which limits the usefulness of these modifications of conjugated polymers for certain applications.^47,48^ However, there are now a number of reactions that meet the above requirements and take place directly on the conjugated polymer backbone or in very close proximity. For this review, we focus exclusively on these functionalisations, as they can alter the electronic and optical properties significantly. We divided the review in sections based on the type of reaction used for these post-polymerisation functionalisations – cycloaddition, oxidation/reduction, nucleophilic aromatic substitution, as well as halogenation and subsequent cross-coupling reaction – and focus our discussion on how these modifications affect the energy levels and optical properties of the polymers.

We further focus on the modification of the chemical composition of the backbone, thereby excluding for example the (reversible) oxidation/reduction of the backbone in doping reactions. In addition, a large body of work has been reported towards the preparation of conjugated ladder polymers, in which adjacent monomers within the conjugated backbone are reacted intramolecularly to provide a rigid link.^49^ These are a fascinating and highly promising class of materials,^50–53^ but we did not consider these reactions here. There are a number of relevant reviews on the preparation of ladder-type conjugated polymers for the interested reader.^54–57^

## Cycloaddition

**Fig. 2:**
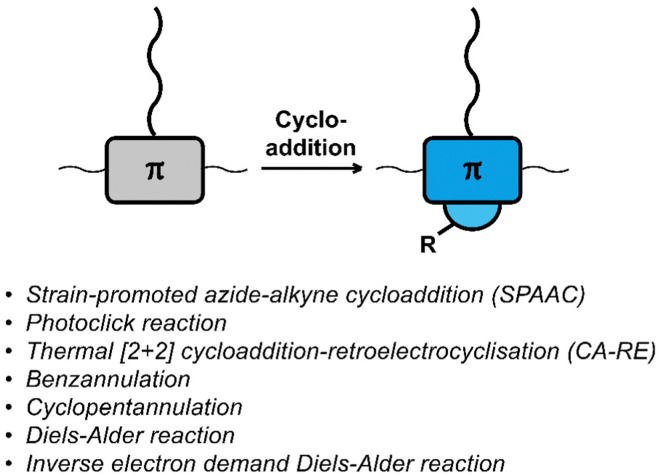
Schematic representation and list of cycloaddition reactions used for post-polymerisation functionalisation of conjugated polymers.

Cycloaddition reactions are frequently used for the functionalisation of polymers and materials.^58,59^ The cycloaddition reactions used for functionalising polymers are usually classified as ‘click reactions’. Click reactions are per definition “modular and orthogonal as well as proceeding under simple and mild reaction conditions while affording high yields of a single product with facile purification”,^60^ which makes click chemistry a powerful approach in polymer chemistry and the development of functional materials.^38,58,59,61–63^ The orthogonality of the reactions and the high yields make them particularly useful for post-polymerisation functionalisation. Fig. 2 provides a list of cycloaddition reactions used for post-polymerisation functionalisation of conjugated polymers discussed in this review.

**Fig. 3:**
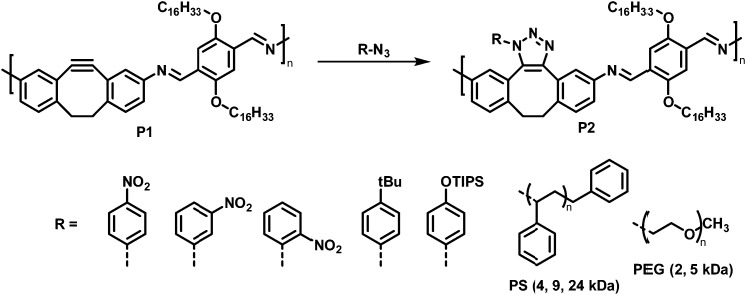
Post-polymerisation functionalisation of polyimine P1 with 5,6-didehydro-11,12-dihydrodibenzo[*a*,*e*]cyclooctene units using azides. Various aryl azides as well as azide-terminated polymers were attached to the polymer backbone *via* SPAAC.^64^

Using a click reaction, Adronov and co-workers demonstrated that the polyimine P1 with 5,6-didehydro-11,12-dihydrodibenzo[*a*,*e*]cyclooctene units can be functionalised *via* strain-promoted azide–alkyne cycloaddition (SPAAC) (Fig. 3, top).^64^ SPAAC has been widely used for the functionalisation or preparation of polymers as well as the formation of supramolecular structures.^65^ Compared to copper-catalysed AAC reactions (CuAAC), SPAAC has lower activation energies and hence requires lower temperatures and proceeds without copper catalysts.^66^ The mild reaction conditions and the non-necessity of a metal catalyst are beneficial for biological systems (for bioorthogonal reactions), enabling *in vivo* applications.^67^ The authors reported that the functionalisation of polymer P1 proceeds quantitatively in just 30 minutes at room temperature using toluene as the solvent and a 1.5-fold excess of azide. The polymer was functionalised with a variety of aryl azides, containing EDGs and EWGs on the aromatic ring (Fig. 3, bottom).

Studying the photophysical properties of the functionalised polymers in solution (THF), P2 showed that the post-polymerisation functionalisation led to small changes in the UV-vis absorption spectrum; the nature of the attached group in P2 has no influence on the UV-vis absorption spectrum. However, in contrast to the alkyne-containing starting polymer P1, the functionalised derivatives did not exhibit any fluorescence. In addition, the authors demonstrated the grafting of azide-terminated polystyrenes with molecular weights of up to 24 kDa, as well as azide-terminated polyethylene glycol monomethyl ether (N_3_-PEG-OMe) of 2 kDa and 5 kDa onto the polyimine under mild reaction conditions (12 h and 60 °C). The coupling of one polymer chain end to the backbone of another polymer can be considered the gold standard in terms of testing the efficacy of any post-polymerisation chemistry, because of the paucity of the polymer end-groups and the sterically challenging environment. Here grafting of the polymers occurred quantitatively in all cases, as indicated by ^1^H NMR and Raman spectroscopy, highlighting the power of the approach. In the case of grafting with N_3_-PEG-OMe, significant changes in the solubility were observed. In contrast to the starting polymer P1, which only exhibited solubility in apolar solvents, these grafted polymers showed good solubility in MeOH (2 kDa N_3_-PEG-OMe) and even water (5 kDa N_3_-PEG-OMe).

**Fig. 4:**
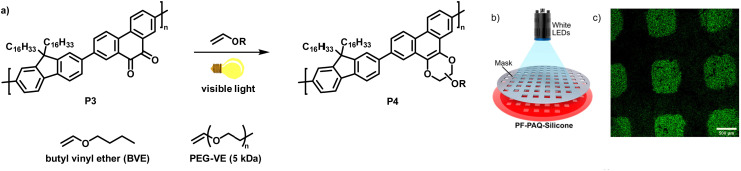
(a) Functionalisation of a 9,10-phenanthrenquinone-containing polymer with vinyl ethers.^68^ (b) Photopatterning of a sample of P3 embedded in a silicon elastomer and exposed to butyl vinyl ether (BVE). (c) Fluorescence microscopy image showing that only the parts of the sample exposed to BVE and light were functionalised. Adapted from ref. 68 with permission, Copyright 2020, American Chemical Society.

In a follow-up work, the authors demonstrated the photoclick functionalisation of a 9,10-phenanthrenquinone-containing conjugated polymer (P3) with electron-rich vinyl ethers (Fig. 4a).^68^ LED illumination of a solution of the polymer and butyl vinyl ether (BVE) resulted in conversion to the cycloaddition adducts P4 in just 5–10 min. Extensive IR spectroscopy experiments verified the quantitative conversion. The authors also studied the influence of the functionalisation on the photophysical properties. They found that the fluorescence intensity of the photoclick product P4 in solution (toluene) was remarkably increased in comparison to the starting material P3. The reaction was also amenable to photopatterning *via* the use of a P3/silicon elastomer blend which was exposed to BVE (Fig. 4b). Only the parts of the samples exposed to BVE and visible light showed emission when examined by confocal fluorescence microscopy, in accordance with the properties of the photoclicked polymer P4 (Fig. 4c). The versatility of the method was further demonstrated by coupling with an end-functionalised polymer (vinyl ether-terminated polyethylene glycol monomethyl ether (PEG-VE)) to afford a water-soluble graft co-polymer.

**Fig. 5:**
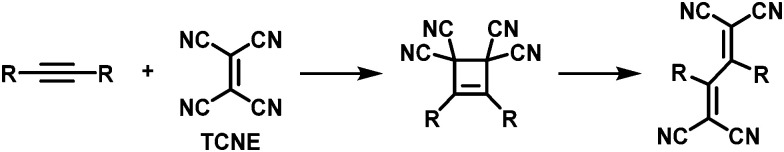
Mechanism of the thermal [2+2] cycloaddition–retroelectrocyclisation (CA–RE) reaction, using tetracyanoethylene (TCNE) as the electron-deficient alkene in this example.

**Fig. 6:**
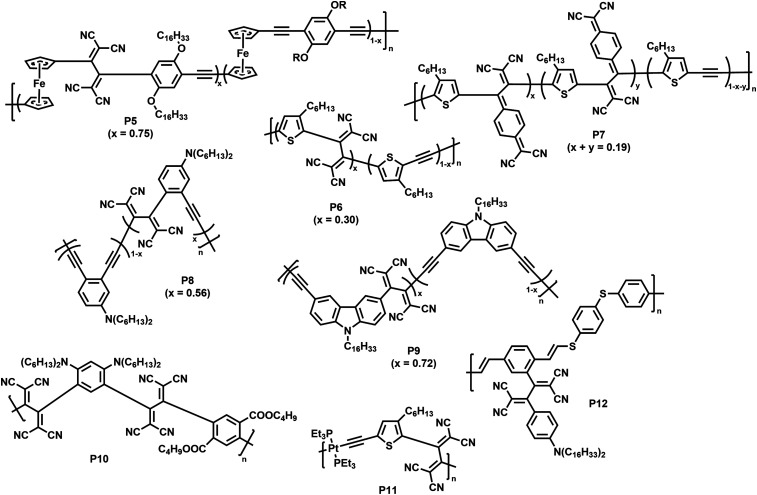
Polymers obtained by post-polymerisation functionalisation *via* CA–RE reaction.^70–75^

Thermal [2+2] cycloaddition-retroelectrocyclisation (CA–RE) reaction is another approach to post-polymerisation functionalisation *via* click chemistry.^69^ In this reaction, electron-rich alkynes in the polymer backbone are reacted with electron-deficient alkenes to form what are usually non-planar donor–acceptor chromophores. In the first step of the reaction, a four-membered ring is formed as an intermediate, which subsequently rearranges by ring-opening leading to the formation of two double bonds (Fig. 5), resulting in the formation of a cross-conjugated donor–acceptor polymer. Strong electron donors need to be present in the polymer backbone in order to achieve sufficient electron density on the alkyne to enable the reaction. Several examples of polymers obtained by this post-polymerisation functionalisation method are shown in Fig. 6 and discussed below. Table 1 provides an overview of their properties.

**Table 1:** Degree of functionalisation and optical and electronic properties of polymers P5–12 obtained *via* CA–RE reactions. Details for the determination of the optical gap (*E*_opt_) and the oxidation and reduction onset potentials (*E*_ox1,onset_ and *E*_red1,onset_) are provided in the footnotes

Polymer	Degree of functionalisation *x*	*E*_opt_ (eV)	*E*_ox1,onset_ (V *vs.* Fc/Fc^+^)	*E*_red1,onset_ (V *vs.* Fc/Fc^+^)	Δ|*E*_ox1,onset_−*E*_red1,onset_| (eV)	HOMO (eV)	LUMO (eV)
P5	0.75	1.46^a^	0.30^d^	−0.86^d^	1.16		
P6	0.30	1.18^b^	0.76^e^	−0.52^e^	1.28	−5.56	−4.28
P7	0.19	1.02^b^	0.92^e^	−0.01^e^	0.93	−5.72	−4.79
P8	0.56	1.00^c^	0.42^d^	−0.61^d^	1.03		
P9	0.72	1.46^c^	0.42^d^	−0.82^d^	1.24	−5.22	−3.98
P10	Quant.	1.40^c^	0.48^f^	−0.62^f^	1.10	−5.42	−4.32
P11	Quant.	1.83^b^	0.63^e^	−0.82^e^	1.45		
P12	n.a.	1.39^c^	1.13^f^	−0.27^f^	1.40	−5.72	−4.32

a From UV-vis absorption spectroscopy in CHCl_3_ solution.

b From UV-vis absorption spectroscopy in *o*-dichlorobenzene solution.

c From UV-vis absorption spectroscopy in CH_2_Cl_2_ solution.

d From cyclic voltammetry (CV) in 0.1 M *n*Bu_4_NClO_4_ in CH_2_Cl_2_.

e From CV in 0.1 M *n*Bu_4_NClO_4_ in CH_3_CN using polymer thin films cast on a glassy carbon electrode.

f From CV in 0.1 M *n*Bu_4_NPF_6_ in CH_2_Cl_2_.

As a first example, Shigehara and co-workers demonstrated the post-polymerisation functionalisation of a ferrocene-containing poly(aryleneethynylene), featuring alkoxy substituents at the arylene units that contribute to the electron-rich character of the polymer.^70^ Functionalisation of the polymer by reaction with tetracyanoethylene (TCNE) in 1,2-dichlorobenzene at 120 °C yielded polymer P5 (with a degree of functionalisation of 75%). The functionalised polymer exhibited charge-transfer bands in the visible region, a characteristic of donor–acceptor chromophores.

Thiophene was reported to be another sufficiently strong backbone donor unit to enable the alkynes to undergo reactions with TCNE for the formation of polymer P6.^71^ As an alternative to TCNE, the authors also functionalised the polymer with 7,7,8,8-tetracyanoquinodimethane (TCNQ), giving polymer P7. As thiophene is a weaker electron donor than ferrocene, the alkynes are less reactive and the reaction was thus performed under microwave irradiation (120 °C, 4 h) to achieve satisfying conversion. The reactions were similarly effective in both cases, with degrees of functionalisation of 30% with TCNE and 19% with TCNQ. The formation of donor–acceptor chromophores in the polymers was confirmed by the appearance of a new broad charge-transfer band (>500 nm) in the visible region of the UV-vis absorption spectrum in solution (measured in *o*-dichlorobenzene) and by the quenching of the fluorescence (measured in CH_2_Cl_2_). Further measurements showed that the electrochemical gap was narrowed, with a lower lying LUMO and an elevated HOMO level.

Michinobu and co-workers studied the functionalisation of a poly(*o*-phenylenebutadiynylene) with TCNE to obtain polymer P8.^72^ They observed the expected shift of the absorption onset to longer wavelengths (in *o*-dichlorobenzene solution) as well as the occurrence of a charge transfer peak in the vis/NIR-region (596 nm). Titration experiments suggested a maximum of 56% functionalisation of the backbone with TCNE. In contrast to the synthesis of P8, the authors reported the appearance of side-reactions when testing the functionalisation of a structurally related poly(*o*-phenyleneethynylene). Similar functionalisation has been reported on a carbazole-containing poly(arylenebutadiynylene), resulting in a narrowed optical gap and the characteristic charge transfer band in the visible region (∼515 nm) for polymer P9 in solution (CH_2_Cl_2_).^73^ A degree of functionalisation of 72% was reported for this polymer.

Poly(arylene ethynylene)s containing both electron-donating dialkylamino groups and electron-withdrawing ester groups are also amenable to functionalisation (P10), with quantitative functionalisation.^74^ A broad charge transfer band was observed at ∼578 nm in solution (CH_2_Cl_2_), which is characteristic for the intramolecular interactions of the dialkylamino and 1,1,4,4-tetracyanobuta-1,3-diene (TCBD) groups. These intramolecular charge transfer interactions also explain the small electrochemical gap of 1.10 eV (determined *via* cyclic voltammetry *vs.* Fc/Fc^+^).

The scope of polymers that can be functionalised *via* the CA–RE reaction was further broadened by Michinobu and co-workers, who functionalised a platinum-containing polyyne in a reaction with TCNE, resulting in polymer P11.^75^ Only one alkyne moiety per repeat unit underwent reaction with TCNE. This indicates that, upon reaction of the first alkyne, the electron density on the other alkyne is lowered, preventing further reaction. The method can also be utilised to functionalise electron rich alkynes attached to the conjugated polymer backbone. For example, alkyne containing poly(vinylene sulfide) was reacted with TCNE, resulting in the formation of donor–acceptor chromophores (P12) with strong intramolecular charge-transfer interactions.^76^

Overall, the CA–RE approach clearly results in significant changes to the optical and electronic properties of the polymers. However, the requirement for very electron-rich starting alkynes and the limited degree of functionalisation in some cases are drawbacks. The loss of backbone planarity may also be undesirable in some cases.

Benzannulation or cyclopentannulation has been shown to be another attractive tool for the synthesis of donor–acceptor polymers by post-polymerisation functionalisation, in particular for the synthesis of polyarylenes. Typically, the preparation of donor–acceptor polyarylenes relies on co-polymerisation of arylene monomers, but this often results in low molecular weight polymers due to steric or electronic influence.^77^ The preparation of polyarylenes *via* a post-polymerisation modification approach can be beneficial, as high molecular weight precursor polymers, synthesised *via* well-studied polymerisation techniques, can be used for the conversion into high molecular weight, low-bandgap copolymers.^78^

**Fig. 7:**
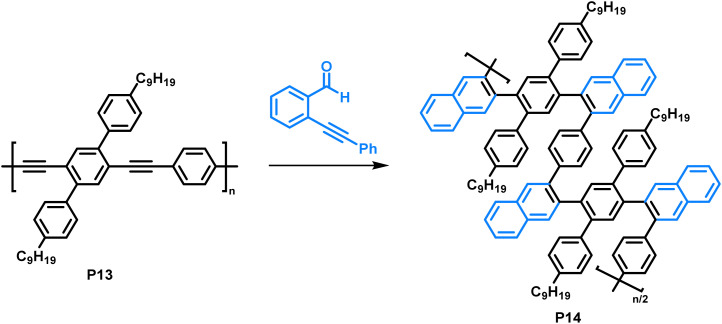
Copper-catalysed benzannulation of poly(phenyleneethynylene) (P13) with *o*-(phenylethynyl)benzaldehyde.^80^

Making use of a copper-catalysed benzannulation reaction,^79^ Dichtel and colleagues synthesised sterically demanding polyarylenes from a poly(phenyleneethynylene) precursor (Fig. 7).^80^ In this post-polymerisation functionalisation, the polymer was reacted with *o*-(phenylethynyl)benzaldehyde (2 equiv.) with the aid of CF_3_COOH (3 equiv.) and Cu(OTf)_2_ (0.05 equiv.). The formation of the benzannulated product was confirmed by a combination of FT-IR and an isotopic labelling NMR study. Samples with ^13^C-enriched alkyne moieties were prepared, and the authors observed a clean shift of the alkyne signals after the benzannulation reaction with no remaining alkyne signals in the ^13^C NMR spectrum, indicating complete conversion. They observed an increase in solubility of the benzannulated product P14 compared to the precursor P13, attributed to steric hindrance in the product. The limited conformational freedom prevents the polymer from adopting a planar conformation. As a result, the behaviour of P14 in solution differs from that of P13, as was observed by size exclusion chromatography. The authors suggest that P14 adopts a more compact structure in solution compared to the non-annulated precursor P13 and is therefore retained longer on the column, despite its higher molecular weight. Molecular dynamics simulations are in accordance with these experimental findings. In UV-vis absorption measurements, a blue shift of the absorption of P14 (*λ*_max_ = approx. 280 nm) of about 140 nm was observed compared to the precursor P13 (*λ*_max_ = approx. 420 nm), which can also be explained by the nonplanarity of the backbone.

**Fig. 8:**
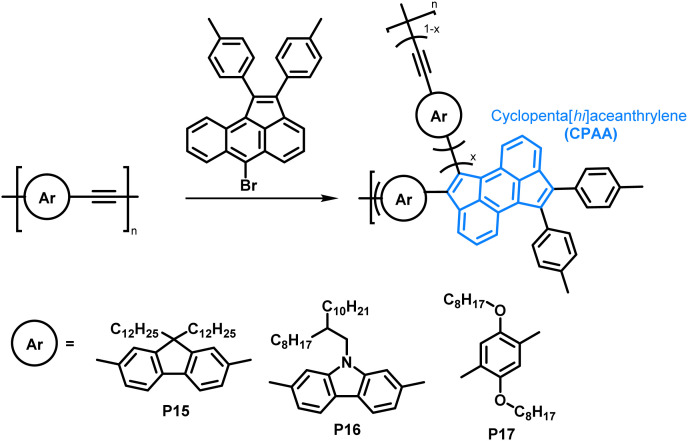
Cyclopentannulation of poly(arylene ethynylene)s as a post-polymerisation functionalisation approach. Polymers containing donor groups of increasing donor strength (from fluorene to carbazole to dialkoxyphenylene).^78^

The scope of the annulation approach has been expanded by Plunkett and colleagues. They earlier reported the palladium-catalysed cyclopentannulation of molecular precursors in order to obtain 1,2,6,7-tetraarylcyclopenta[*hi*]aceanthrylenes.^81^ Adapting this reaction for the functionalisation of acetylene-containing conjugated polymers (Fig. 8), an excess of aryl bromide was used in order to achieve a high degree of functionalisation, giving polymers P15–17.^78^ Analysis of polymers enriched with ^13^C (analogues of P15) by ^13^C NMR spectroscopy showed that unreacted alkynes were still present after functionalisation, but ”near complete conversion” was reported. The incomplete functionalisation was attributed to the reduced access to the polymer backbone, owing to a more folded structure upon functionalisation. The occurrence of side reactions was ruled out as a possible explanation, as the alkyne functionalities would be consumed likewise. GPC data showed similar trends as reported by Dichtel *et al.*; upon annulation the conformation of the polymer changes significantly to a more compact structure, resulting in longer retention times than observed for the precursor polymer. Formation of the cyclopenta[*hi*]aceanthrylene unit bathochromically shifted the thin film absorption onset from approx. 600 nm in the precursor to above 800 nm in the functionalised polymer. The donor strength of the respective aromatic moiety (increasing from fluorene to carbazole to dialkoxyphenylene) did not affect the optical and electronic properties of the polymers significantly (Table 2), with all three polymers, P15, P16 and P17, having very similar optical bandgaps of ∼1.5 eV.

**Table 2:** Optical and electronic properties of polymers P15–17 prepared *via* cyclopentannulation. The absorption onset wavelengths (*λ*_onset_) were determined measuring thin films of the polymers and converted to give the optical band gaps (*E*_g_ (opt.)). *E*_ox1,onset_ and *E*_red1,onset_ were obtained from CV measurements using polymer thin films (in 0.1 M *n*Bu_4_NPF_6_ in CH_3_CN)

Polymer	*λ*_onset_ (nm)	*E*_g_ (opt.) (eV)	*E*_ox1,onset_ (V *vs.* Fc/Fc^+^)	*E*_red1,onset_ (V *vs.* Fc/Fc^+^)	Δ|*E*_ox1,onset_−*E*_red1,onset_| (eV)	HOMO (eV)	LUMO (eV)
P15	817	1.52	0.70	−1.37	2.07	−5.50	−3.43
P16	821	1.51	0.64	−1.39	2.03	−5.44	−3.41
P17	824	1.50	0.61	−1.42	2.03	−5.41	−3.38

**Fig. 9:**
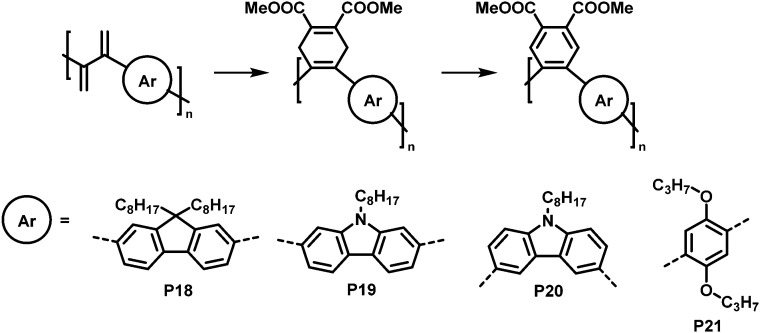
Post-polymerisation functionalisation of a diene-containing polymer *via* Diels–Alder reaction. Four different aromatic units were incorporated in the main chain of the conjugated polymer. After Diels–Alder reaction, the polymers were rearomatized in an additional step.^82^

An interesting cycloaddition approach reported by Hayashi and colleagues involved the reaction of diene containing cross-conjugated precursor polymers with a dienophile *via* Diels–Alder reaction (Fig. 9).^82^^1^H NMR spectra showed full conversion of the diene in the polymer backbone (by the disappearance of the signals of the diene protons). Similarly, full conversion was also confirmed for the subsequent rearomatisation step. The obtained all-(*Z*)-alkene- and *ortho*-arylene-containing polymers exhibited remarkable Stokes shifts (∼150 nm) in solution (THF), attributed to the π–π interactions between the arylene moieties.

**Fig. 10:**
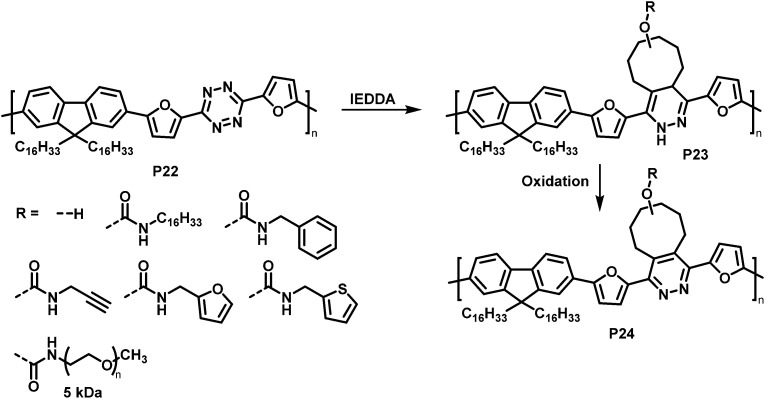
Post-polymerisation functionalisation of a tetrazine-containing polymer *via* inverse electron demand Diels–Alder (IEDDA) reaction and subsequent oxidation to rearomatise the unit.

Similarly, tetrazine containing polymer P22 is amenable to inverse electron demand Diels–Alder (IEDDA) reactions with a variety of cyclooctenes (Fig. 10).^83^ The reaction takes place rapidly at room temperature in solution, using only a small excess of the respective electron-rich dienophile. Using seven different *trans*-cyclooctene derivatives (see R in Fig. 10), the authors exemplified how conveniently different groups can be quantitatively introduced *via* this approach. It needs to be noted that the cycloaddition products P23 are always a mixture of two regioisomers. In a following step, the 1,4-dihydropyridazine rings were oxidised using 2,3-dichloro-5,6-dicyano-1,4-benzoquinone (DDQ) to rearomatize and hence restore conjugation in polymer P24. The emission intensity in solution (THF) was substantially increased upon functionalisation with *trans*-cyclooctenes.

## Oxidation and reduction

**Fig. 11:**
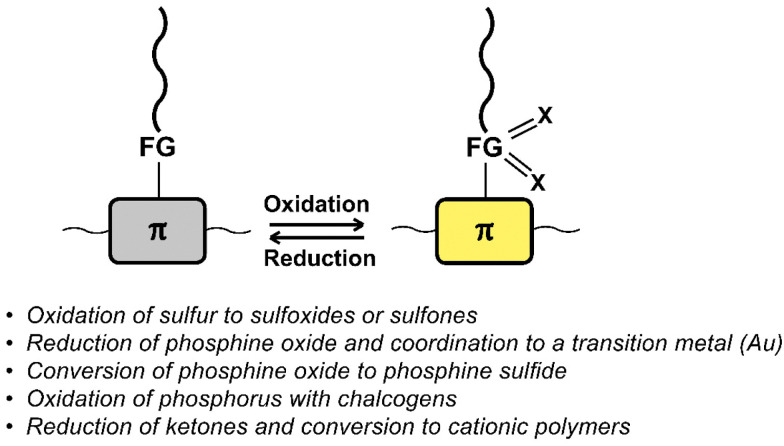
Schematic representation and list of oxidation and reduction reactions used for post-polymerisation functionalisation of conjugated polymers.

**Fig. 12:**
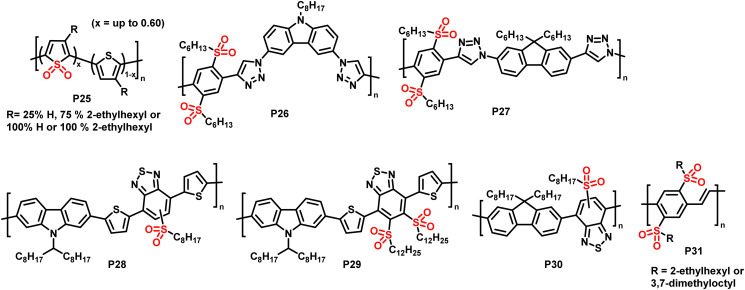
Sulfone-substituted polymers P25–31 prepared *via* post-polymerisation oxidation of sulfur atoms in polythiophenes and alkylthio-substituted polymers.^84–88^

Fig. 11 provides a list of oxidation and reduction reactions used for post-polymerisation functionalisation of conjugated polymers discussed in this review. The first reaction – the oxidation of sulfur atoms in the backbone or in functional groups attached to the backbone to the corresponding sulfoxides or sulfones – has been shown to be a particularly fast and selective approach for post-polymerisation functionalisation of conjugated polymers.^84–88^ The approach is especially useful to obtain polymers with significantly altered properties from a starting polymer in just one step, as the electron-donating sulfur is transformed into electron-withdrawing groups by the oxidation. Fig. 12 shows sulfone-substituted polymers P25–31 obtained by this approach and Table 3 provides an overview of the reported optical and electronic properties. By making use of this post-polymerisation oxidation, n-type conjugated polymers can be obtained that may not be accessible by polymerisation of the oxidised monomer.^88^ Using this approach, a continuous junction design for organic electronic devices relying on post-processing oxidation of electron-donor polymer thin films has been proposed, as discussed in more detail below.^85^

In 2014, Campos and colleagues reported the use of Rozen's reagent (HOF·CH_3_CN) for the oxidation of thiophene in order to obtain thiophene-1,1-dioxide (TDO)-containing polymers P25.^84^ The reaction occurred rapidly at room temperature, and 60% of the polymeric thiophene was converted to TDO by reaction with 4 equiv. of Rozen's reagent. The addition of more than 4 equiv. of the reagent did not increase the extent of oxidation, but rather enhanced the occurrence of side reactions. The absorption onset of the polymer (measured in CHCl_3_ solution) was shifted significantly to longer wavelengths by the oxidation, and ultraviolet photoelectron spectroscopy (UPS) measurements of thin films found that both the HOMO and LUMO energy levels were lowered, with a larger influence on the latter. The electron accepting character of the oxidised polymer was further confirmed by its ability to effectively quench the fluorescence of poly(3-hexylthiophene) (P3HT). The authors also demonstrated that the backbone oxidation could be performed in the presence of potentially reactive end groups, such as hydroxy groups, opening up the possibility of further end-group modification.

In the same year, Fröhlich, Glöcklhofer and co-workers introduced dimethyldioxirane (DMDO) as a suitable oxidising agent for post-polymerisation oxidation.^86^ DMDO is a versatile and mild reagent that gives acetone as the only by-product, facilitating purification after the modification. Excellent efficiency and selectivity further support the use of this reagent in post-polymerisation functionalisation reactions. Initially, they used DMDO for the oxidation of polymers prepared by microwave-assisted CuAAC.^86^ A series of monomers containing selenium, tellurium and sulfur were prepared and polymerised, and the sulfur-containing polymer was fully converted to the sulfone-containing substituted polymer P26. Measuring the polymers in CH_2_Cl_2_ solution, no change in the absorption wavelengths was observed in this case, but the emission was red shifted by ∼80 nm, from blue to yellow.

**Table 3:** Optical and electronic properties of P25–27 and P31, determined by UV-vis absorption spectroscopy and CV of thin films (if not stated otherwise). CV measurements were performed in 0.1 M *n*Bu_4_NPF_6_ in CH_3_CN

Polymer	*E*_g_ (opt.) (eV)	*E*_ox1,onset_ (V *vs.* Fc/Fc^+^)	*E*_red1,onset_ (V *vs.* Fc/Fc^+^)	HOMO (eV)	LUMO (eV)
P25^a^	1.76^e^	—	—	−5.79^b^	−4.03^b^
P26	3.2	0.79	−2.4^c^	−5.59	−2.4
P27	3.2	1.17	−2.0^c^	−5.97	−2.8
P31^d^	2.47	—	−1.14	−6.13 (opt.)	−3.66

a *R* = 75% H, 25% 2-ethylhexyl.

b Determined *via* ultraviolet photoelectron spectra (UPS).

c Estimated based on *E*_ox1,onset_ and *E*_g_ (opt.)

d R = 2-ethylhexyl.

e Optical gap (*E*_opt_), from UV-vis absorption spectroscopy in CHCl_3_ solution.

OPV devices usually rely on the separation of tightly bound electron–hole pairs, so called excitons, at the interface of a donor and an acceptor material.^14^ In order to increase the interface area and, thus, the efficiency of the charge separation, most OPV devices make use of the bulk heterojunction (BHJ) concept, where the two materials are mixed to form a bicontinuous network. A new concept was proposed by Fröhlich and colleagues in 2015, which is based on post-processing oxidation of alkylthio-substituted donor polymers for the *in situ* preparation of acceptor polymers.^85^ Treating donor polymer thin films with DMDO solution was suggested to result in an oxidation gradient within the films, which would gradually change the electronic character by the conversion of electron-donating alkylthio groups (+M effect) into electron-withdrawing sulfoxide and sulfone groups (−M effect). One of the advantages of such a continuous junction would be that only one material needs to be prepared by polymerisation, which significantly lowers the synthetic effort. The authors prepared polymers P26 and P27 in order to test the conversion and to compare the properties to those of the starting polymers.^85^ Cyclic voltammetry measurements confirmed that the oxidised polymer exhibited lower lying HOMO and LUMO energy levels. However, the energy offset of the LUMO levels was too small to ensure efficient exciton dissociation with these polymers, and the low degree of polymerisation combined with the presence of triazole units in the backbone limited charge carrier mobility. In some more recent follow-up work, the use of DMDO to alter the photophysical properties of alkylthio-substituted conjugated polymers was demonstrated in donor–acceptor type materials (P28–30) as well as alkylthio-substituted poly(*p*-phenylene vinylene)s P31.^87,88^

**Fig. 13:**
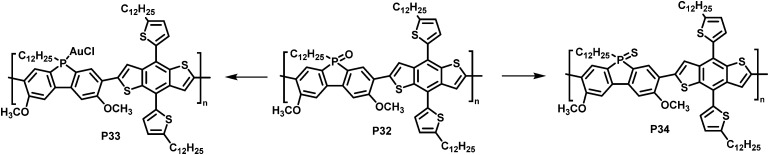
Modification of phosphafluorene oxide-containing polymer P32.^89^

In a different approach, Rupar and colleagues reported the post-polymerisation modification of phosphafluorene oxide-containing copolymers (Fig. 13).^89^ The initially prepared donor–acceptor type copolymer P32 could undergo three different modifications at the phosphorus centre: (i) reduction of the P-centre with HSiCl_3_, although the resulting polymer rapid re-oxidized in presence of air, (ii) quantitative reduction and coordination to a transition metal such as gold (AuCl), enabling air-stability of the resulting polymer P33, and (iii) quantitative conversion of the phosphine oxide to phosphine sulfide with Lawesson's reagent, giving polymer P34. Gold complexation resulted in a significant change in optical and electronic properties, compared to the parent P32, whereas changing the phosphine oxide to the phosphine sulfide had a more subtle effect (Table 4).

**Table 4:** Optical and electronic properties of P32–34

Polymer	*λ*_max,ab_ a (nm)	*λ*_max,em_ a (nm)	*λ*_onset_ a (nm)	LUMO^b^ (eV)	HOMO^b^ (eV)	*E*_g,CV_ b (eV)
P32	465	521	538	−2.99	−5.56	2.57
P33	530	550	555	−3.43	−5.23	1.80
P34	466	520	534	−3.03	−5.46	2.43

a From UV-vis absorption spectroscopy in CH_2_Cl_2_ solution.

b From CV measurements in 0.1 M *n*Bu_4_NClO_4_ in CH_3_CN measuring polymer thin films cast onto the working electrode.

**Fig. 14:**
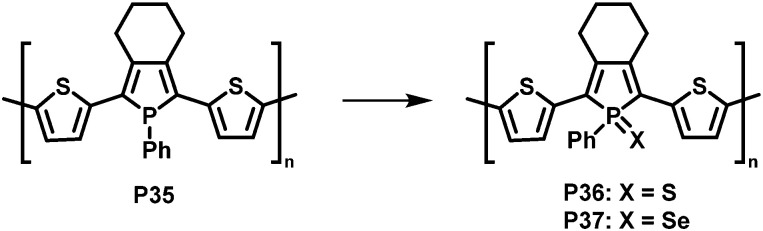
Modification of phosphole-containing polymers by oxidation with chalcogens such as sulfur and selenium.^90^

In a related, albeit inverted approach, Réau *et al.* prepared phosphole-containing polymer P35 (Fig. 14),^90^ which was oxidised with chalcogens such as sulfur and selenium, giving polymers P36 and P37. ^31^P MAS NMR and SEM-EDX confirmed the almost quantitative oxidation of the phosphorus-centres with sulfur. Reaction with sulfur or selenium was found to influence the optical and electronic properties of the conjugated polymers, enabling potential sensing applications.

**Fig. 15:**
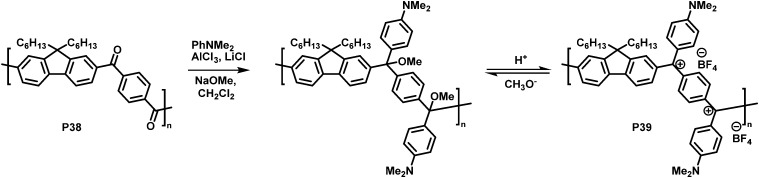
Conversion of a cross-conjugated polymer into a fully conjugated cationic polymer.^91^

In another interesting approach, Chiechi and co-workers reduced the cross-conjugated polymer P38, initially yielding a non-conjugated polymer with ether groups. Subsequent treatment with acid formed a charged, fully conjugated polymer P39 (Fig. 15), which could be switched reversibly.^91^ Very large changes in the absorption onset of the polymer (in CH_2_Cl_2_ solution) were observed upon modification. The absorption onset shifted from 3.26 eV (380 nm) for cross-conjugated P38 to 1.55 eV (800 nm) for P39. Subsequent work on related materials demonstrated how they could be fabricated into proof-of-concept bilayer solar cells from aqueous formic acid.^92^

## Nucleophilic aromatic substitution (S_N_Ar)

**Fig. 16:**
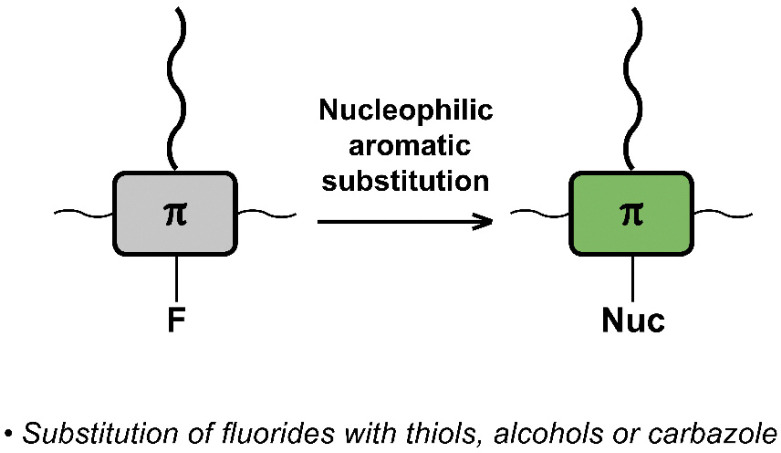
Schematic representation of nucleophilic aromatic substitution used for post-polymerisation functionalisation of conjugated polymers. All reactions in this section relied on the substitution of fluorides, but various different nucleophiles were used, such as thiols, alcohols and carbazole.

**Fig. 17:**
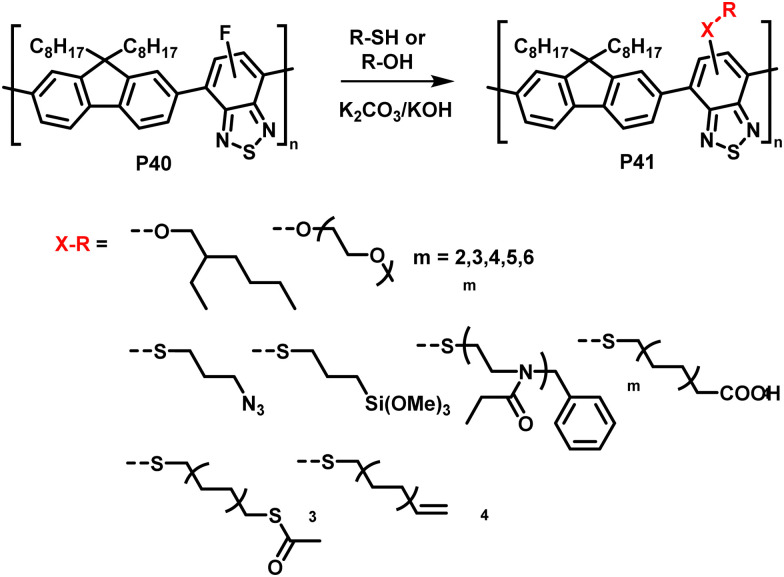
Nucleophilic aromatic substitution of fluorinated, fully conjugated F8FBT.^93,95^

The interchange of functional groups on the polymer backbone is another approach to introduce varying functionality (Fig. 16), with recent work demonstrating the usefulness of nucleophilic aromatic substitution (S_N_Ar) reactions in post-polymerisation functionalisation.^93^ Electron deficient aromatics containing halide leaving groups are particularly amenable to S_N_Ar reactions, with fluorinated leaving groups being particularly active, due to their strong electron withdrawing effect. For example, fluorinated 2,1,3-benzothiadiazole has been shown to be active to S_N_Ar reactions under mild conditions.^94^ Incorporation of such monomers into a conjugated backbone allows the displacement to occur post polymerisation. Thus, reaction of fully conjugated poly(9,9-dioctylfluorene-*alt*-5-fluoro-2,1,3-benzothiadiazole) (F8FBT) P40 with a series of thiols and alcohols in the presence of base affords polymers P41 with different X–R groups (Fig. 17). Due to the change from an electron withdrawing fluorine substituent to a donating alkoxy or thioether group, substitution was shown to have significant effects on the optical gap. The reaction conditions even enabled introduction of sidechains containing orthogonal functional groups, which can be used for further functionalisation. For example, azide-containing substituents were readily introduced *via* S_N_Ar, and semiconducting polymer nanoparticles were prepared of the resulting polymers. Surface azides were then shown to undergo CuAAC or SPAAC, reactions widely used in materials chemistry and bioconjugation.^45,59^ The authors showed that the degree of substitution can simply be varied by the amount of nucleophile present, as indicated by ^1^H NMR and UV-vis absorption spectroscopy. Remarkably, this reaction also enables the preparation of multi-functionalised polymers in a one-pot procedure, for instance polymers containing not only azide groups but also carboxylic acid and silane groups.

**Fig. 18:**
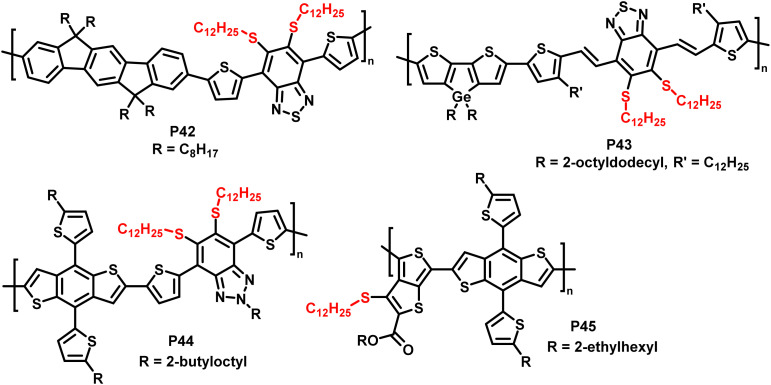
Polymers obtained by nucleophilic aromatic substitution, showing that the substitution approach is amenable to polymers with a variety of acceptor units such as fluorinated benzotriazole and thieno[3,4-*b*]thiophene.^93^

In addition, the authors have shown the substitution of two fluorides on 5,6-difluoro-2,1,3-benzothiadiazole units (P42 and P43 in Fig. 18), using an excess of thiol. Furthermore, the reaction has also been proven to be feasible on other fluorinated acceptor units, such as alkylated 5-fluoro-1,2,3-benzotriazole (P44 in Fig. 18), which is less electron accepting in nature, and fluorinated thieno[3,4-*b*]thiophene (P45 in Fig. 18). In both cases, complete substitution was reported.

In a later work, the functionalisation of fluorinated F8FBT with a series of ethylene glycol oligomers as well as polyethylene glycol (PEG) by S_N_Ar was demonstrated.^95^ Typically, conjugated polymers are hydrophobic and therefore insoluble in highly polar solvents. However, for some applications it is desirable that the materials feature hydrophilic functionalities, which renders them soluble in polar, greener solvents such as water and alcohols,^96^ and explains the authors’ interest in introducing hydrophilic (poly)ethylene glycol side chains. Moreover, the increased wettability of polymers substituted with such side chains enables efficient contact with cell tissue.^97^ The hydrophilicity of the polymers was gradually increased with increasing length of the ethylene glycol sidechains, as indicated by contact angle measurements and solubility tests. The photophysical properties are, however, not influenced by the increasing length of ethylene glycol chains.^95^

**Fig. 19:**
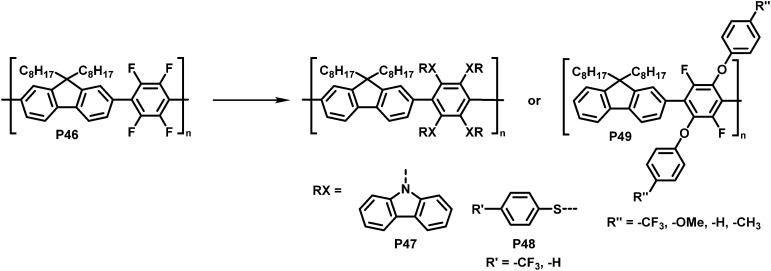
Functionalisation of tetrafluorophenylene-containing polymers *via* S_N_Ar with *S*-, *O*- and *N*-nucleophiles.^98^

Inagi and colleagues broadened the scope of polymer backbones that can undergo S_N_Ar reactions.^98,99^ They prepared a tetrafluorophenylene-fluorene copolymer P46 and subsequently functionalised it with a variety of *S*-, *O*- and *N*-nucleophiles to yield polymers P47–49 (Fig. 19), facilitating control of the photophysical properties. Similarly, intramolecular reactions of amine functionalised co-polymers have been reported.^100^

**Fig. 20:**
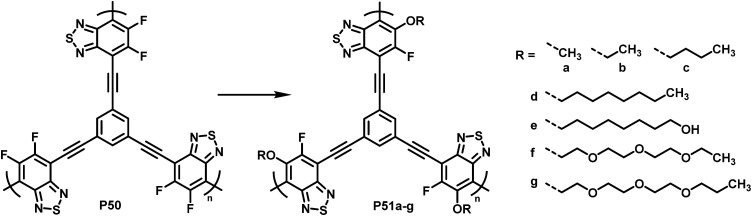
BT-based polymers for photocatalytic hydrogen evolution after functionalisation with various sidechains R.^101^

To demonstrate what a helpful tool for quick modification of polymer characteristics post-polymerisation functionalisation can be, Chen *et al.* used nucleophilic aromatic substitution to improve the performance of a conjugated polymer in photocatalytic H_2_-evolution.^101^ Various substituents of different hydrophilicity, ranging from methoxy groups to glycol chains, were introduced into hyperbranched polymer P50 (Fig. 20). The authors showed that by increasing the affinity to water, the hydrogen evolution rate (HER) increases. This was demonstrated by comparing contact angles and the HER. Compared to the starting polymer containing difluorinated BT units (P50), the HER was increased almost 2.5-fold, from 126 to 311 μmol h^−1^ for P51g with a glycol chain.

## Halogenation & other electrophilic aromatic substitution (S_E_Ar) and Pd-catalysed cross-coupling

**Fig. 21:**
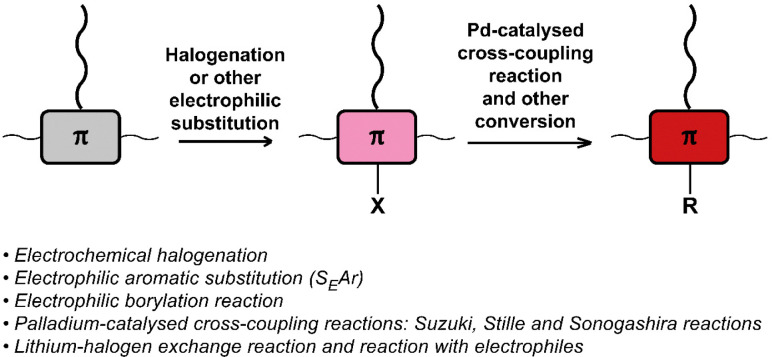
Schematic representation and list of (i) halogenation and (other) electrophilic substitution reactions used for post-polymerisation functionalisation and (ii) Pd-catalysed cross-coupling reactions and other conversion of halogenated conjugated polymers.

The halogenation of conjugated backbones has been demonstrated on a wide variety of polymers *via* both electrophilic and nucleophilic approaches. Halogenation has the potential to alter the optical and electronic properties of the polymer, but it also allows for many subsequent functionalisation reactions. Fig. 21 provides a list of halogenation/electrophilic substitution and cross-coupling reactions used for post-polymerisation functionalisation of conjugated polymers that are discussed in this review.

One of the earliest reports utilised the inherent electroactive nature of the conjugated backbone, showing that electrochemical oxidation of various conjugated polymers in the presence of halides can result in backbone halogenation.^102^ Interestingly, the reaction was performed on the solid film, itself prepared *via* electrochemical polymerisation. For example, film oxidation in a 0.1 M Et_4_NCl/CH_3_CN solution resulted in 100% chlorination of poly(3-methylthiophene) and polythiophene after repeated oxidations. Other halides were also explored for the substitution on poly(3-methylthiophene); 50% bromination was achieved with Bu_4_NBr under the same conditions. The authors also tested iodination but found that the reactivity of the iodide nucleophile is not sufficient, in agreement with the nucleophilicity of halides in aprotic solvents (Cl^−^ > Br^−^ > I^−^). Moreover, alkoxylation with 3-bromopropanol was shown to be feasible with approximately 25% substitution on poly(3-methylthiophene). The same approach was also used for the chlorination of poly(3-(4-fluorophenyl)thiophene) on the conjugated backbone.^103^ Co-polymers of thiophene and fluorene are also amenable to electrochemical bromination (Br introduced per thiophene unit: 1.1) and chlorination (Cl introduced per thiophene unit: 1.7).^104^ Again, attempts to iodinate the polymer were unsuccessful, further confirming the insufficient nucleophilicity of iodide. Lastly, the presence of a Lewis acid has been shown to facilitate such reactions, *via* shifting of the oxidation onset potential of the conjugated polymer.^105^ This was shown to be effective for anodic chlorination of P3HT, poly(*p*-phenylene) and poly(9,9-dioctylfluorene) using an AlCl_3_/acetonitrile system, with high current efficiency and high degrees of chlorination (79% for P3HT at optimum conditions).

**Fig. 22:**
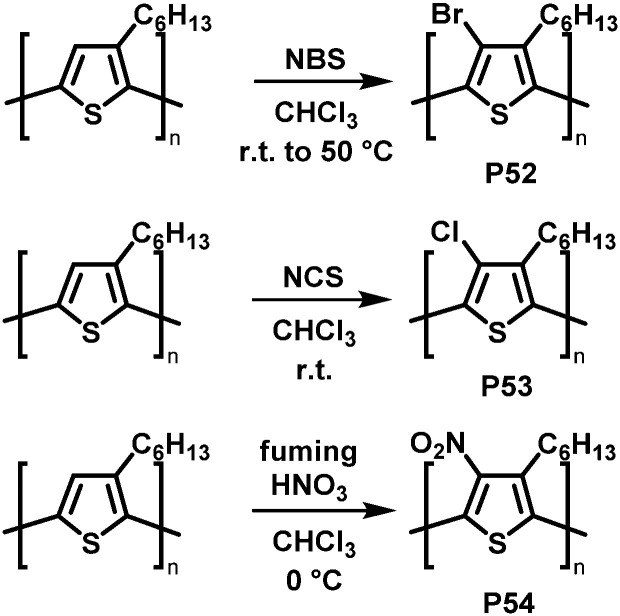
Selected examples of P3HT functionalisation.^106^

Backbone functionalisation under electrophilic aromatic substitution (S_E_Ar) conditions has also been successful, as reported for P3HT by Holdcroft and co-workers in early 2001.^106^ Treatment with *N*-bromosuccinimide (NBS), *N*-chlorosuccinimide (NCS), or fuming nitric acid (HNO_3_) resulted in 100% substitution in the 4-position with bromo (P52), chloro (P53) and nitro groups (P54) produced in high yield (Fig. 22). Functionalisation resulted in a widening of the optical gap in all cases, attributed to the large size of the substituents causing backbone twisting. In follow-up work, they applied this electrophilic substitution approach to poly(2,5-dihexyloxy-1,4-phenylenevinylene) (DHO-PPV).^107^ In this case, the reaction occurred not only on the phenylene units but also on the vinylene bridges. ^1^H NMR spectroscopy indicated that 30% of the reaction with NBS occurs at the bridges, the remaining 70% at the phenylene units. For chlorination, similar results were obtained: 22% of the reaction with NCS occurs at the vinylene bridges, 78% at phenylene units. The reaction at the vinylenes leads to a rupture of the conjugation and therefore decreases the conjugation length. It was demonstrated that by modifying the ratio of reagent (NBS or NCS) to vinylene units it is possible to control the absorption and emission maximum wavelengths as well as the fluorescence efficiency.

**Fig. 23:**
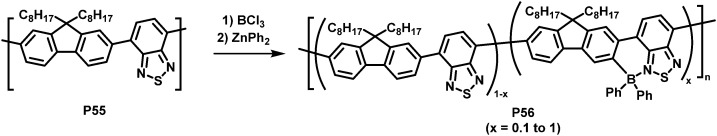
Introduction of heteroatoms as a strategy to alter the optical and electronic properties of conjugated polymers.^108^

An unusual electrophilic substitution of the polymer backbone has been reported by Ingleson and co-workers who utilised an electrophilic borylation reaction of F8BT (P55) with BCl_3_, followed by reaction with diaryl zinc to prepare near-infrared emitting polymer P56 (Fig. 23).^108^ The electron-accepting boryl group adjacent to the 2,1,3-benzothiadiazole unit results in the formation of a dative B–N bond. The resulting planarization of the conjugated backbone in combination with the electron withdrawing effect of the introduced functional group resulted in a significant red shift of the emission of the functionalised polymers (in solution and thin film). The percentage of borylation was readily controlled by varying the equivalents of BCl_3_ added in the synthesis (*x* = 0.1 to 1 in P56). The solid-state emission maximum of the fully borylated polymer was redshifted by about 200 nm to a wavelength of 757 nm and a photoluminescence quantum yield of 11% was determined. The electrochemical gap becomes considerably smaller by the C–H borylation, from ∼2.60 eV to 1.94 eV (determined by CV in 0.1 M *n*Bu_4_NPF_6_ in CH_2_Cl_2_). Those characteristics combined with excellent stability to protic species suggest that the polymers are applicable for *in vivo* biological imaging. The authors prepared encapsulated nanoparticles and confirmed excellent photophysical properties and stability as well as good biocompatibility.^109^

**Fig. 24:**
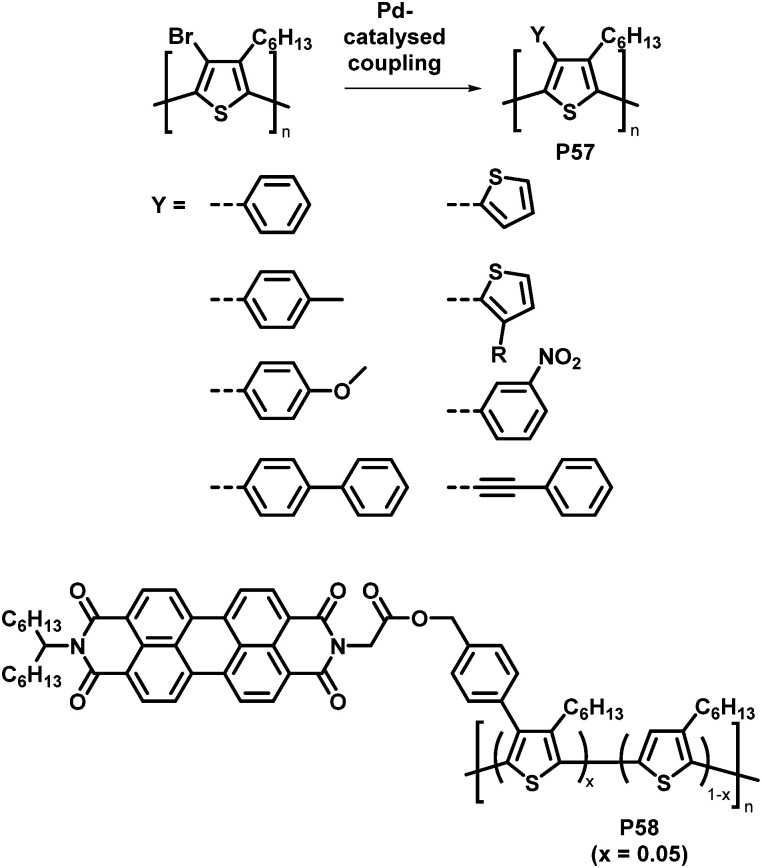
Products of Pd-catalysed coupling reactions of halogenated P3HT with different aromatic groups Y (top)^112^ and P3HT-bearing perylene diimide (PDI) units introduced by Suzuki cross-coupling (bottom).^113^

An attractive feature of backbone bromination is the ability to subsequently engage in palladium-catalysed cross-coupling reactions, such as Suzuki, Stille and Heck reactions. Holdcroft and colleagues demonstrated this with their brominated P3HT, resulting in a number of functionalised derivatives of P3HT (P57) in excellent yields.^110^ The introduction of aromatic groups (Y in Fig. 24) resulted in a remarkably increased fluorescence efficiency in the solid state (9–22%) compared to unfunctionalised P3HT (1.6%) and a red shift in the absorption and emission maxima.^111,112^ Moreover, with the use of a Suzuki coupling reaction, perylene diimide (PDI) containing P3HT derivative P58 was realised.^113^ This polymer was used to assist in templated crystal growth of free PDI, demonstrating a useful approach for obtaining supramolecular structures.

**Fig. 25:**
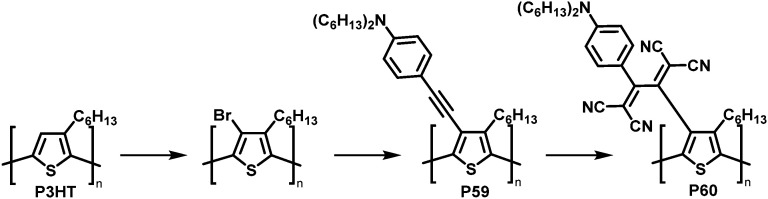
Post-polymerisation functionalisation of P3HT: halogenation followed by Stille coupling for the synthesis of P59 and subsequent reaction with TCNE giving P60.^114^

The combination of consecutive post-polymerisation reactions demonstrates the power of the approach. For example, bromination and subsequent Stille reaction of P3HT affords polymer P59 (Fig. 25).^114^ This was then used in a [2+2] CA–RE reaction with TCNE, a reaction discussed in detail in the section on cycloaddition above, resulting in an electron-accepting TCBD unit (P60). Interestingly electrochemical reduction of P60 created stable poly(anionic radical)s. Evidence for the stability at room temperature of the poly(anionic radical)s was presented in the form of electron spin resonance (ESR) measurements.

The utility of brominated P3HT was further highlighted by Swager and co-workers, who used it for a lithium–halogen exchange reaction.^115^ A nearly quantitative exchange was observed and the resulting lithiated P3HT was reacted with a variety of functional electrophiles. Thus, a broad range of functionalised P3HT was obtained, including polymers with functional groups such as ketones, alcohols (primary and secondary) and azides, as well as silanes and fluoride moieties. The functional groups enabled the authors to perform further reactions, such as a click reaction of the azides with alkynes.

**Fig. 26:**
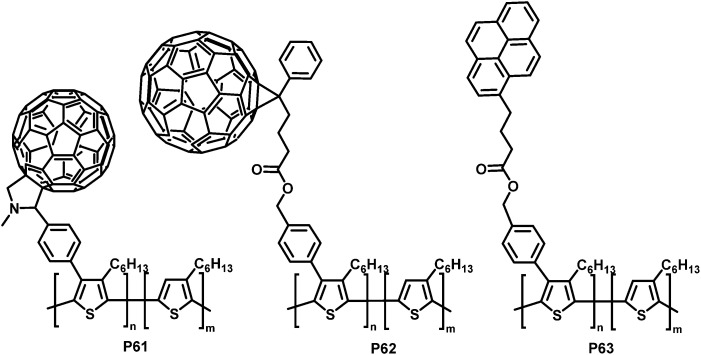
P3HT functionalised with carbon moieties that are able to interact with the fullerene acceptor materials or nanotubes and can therefore improve device performance.^120–122^

Much of the work in conjugated backbone functionalisation has been driven by the challenge of creating bicontinuous networks of electron-rich donor materials (most commonly a conjugated polymer such as P3HT) and electron-deficient acceptor materials (such as fullerenes) for BHJ solar cells.^14,116,117^ Often, due to poor miscibility, the interfacial area between donor and acceptor is small, which can cause poor charge separation and transport.^118,119^ Yang and co-workers addressed this issue by functionalising P3HT with side-chains bearing groups that can improve the contact between the donor and acceptor material and decrease macrophase separation of the two materials.^120–122^ They prepared a soluble P3HT derivative bearing fullerenes (P61 in Fig. 26) *via* a post-polymerisation functionalisation route.^121^ P3HT was brominated with NBS with a degree of functionalisation between 12 and 20% in the first step, followed by a Suzuki coupling reaction with 4-formylphenylboronic acid and a subsequent Prato reaction with C_60_, yielding C_60_-Ph-P3HT (P61) (100% of Br converted in the coupling reaction). The fluorescence in solution is almost fully quenched (compared to P3HT or P3HT:C_60_ blend), indicating that electron transfer is readily occurring. However, X-ray powder diffraction (XRD) studies suggested the lamellar packing of P3HT was slightly perturbed by the presence of the tethered fullerene.

Similarly, PCBM ([6,6]-phenyl-C_61_-butyric acid methyl ester) was introduced to P3HT using the same post-polymerisation functionalisation strategy and was shown to have a similar effect on the fluorescence.^120^ Here it was found that the bicontinuous interpenetrating network morphology of a P3HT:PCBM blend was improved by adding PCBM-Ph-P3HT (P62 in Fig. 26) to the blend, as indicated by AFM morphology studies. Fabrication of photovoltaic devices indicated an increase in PCE by 12% at the optimum ratio (1 wt% PCBM-Ph-P3HT), albeit with relatively low PCE in all cases (Table 5).

**Table 5:** Device data for bulk heterojunction polymer solar cells (BHJ-PSCs) composed of P3HT and PCBM with different amounts of PCBM-Ph-P3HT (P62)^120^

Composition of photoactive layer^a^	PCBM-Ph-P3HT (wt%)	*V*_OC_ (V)	*J*_SC_ (mA cm^−2^)	FF (%)	PCE (%)
0 : 1 : 0.8	0	0.62	8.93	0.54	3.03
0.009 : 1 : 0.8	0.5	0.60	9.82	0.54	3.13
0.018 : 1 : 0.8	1	0.62	9.92	0.55	3.40
0.036 : 1 : 0.8	2	0.63	9.69	0.54	3.28

a PCBM-Ph-P3HT : P3HT : PCBM = *x* mg : 1 mg : 0.8 mg.

SWCNTs (single-walled carbon nanotubes) are another interesting electron acceptor material that has been investigated as an alternative to fullerene-based acceptors.^123^ Yang *et al.* prepared pyrene-functionalised P3HT derivative P63 (Fig. 26, 15% substitution) by post-polymerization modification and observed increased interactions with SWCNTs as a result of the introduction of the pyrene units.^122^

**Fig. 27:**
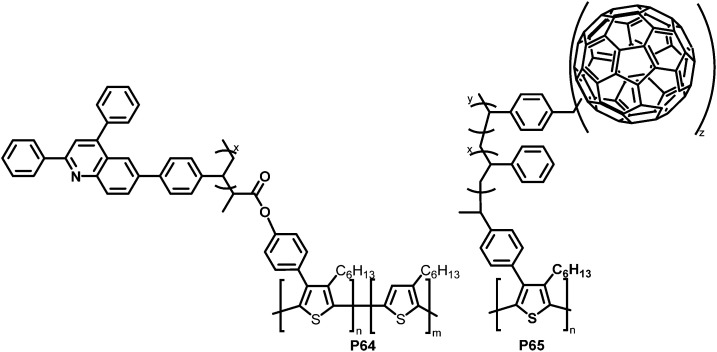
Brush-type graft-copolymers, obtained *via* Suzuki coupling followed by radical polymerisation techniques.^124–126^

With the same intention of improving the chemical compatibility between donor and acceptor material, Hadziioannou and colleagues investigated a grafting-onto strategy to obtain brush-type copolymers by post-polymerisation functionalisation of P3HT.^124^ P3HT was brominated and propionyl bromide groups were introduced as polymerisation initiators *via* Suzuki coupling and further modification. A quinoline containing vinyl monomer (SDPQ) was then polymerised onto the polymer under atom transfer radical polymerisation (ATRP) conditions. The authors found that upon addition of the resulting copolymer P64 (Fig. 27) to a blend of P3HT:PSDPQ, the surface roughness and phase separation were reduced significantly, therefore improving the overall morphology.

Using a related post-polymerisation functionalisation approach, the grafting of fullerene containing sidechains onto P3HT was presented as another strategy to prevent the undesired large-scale phase separation and the ensuing reduction of the donor–acceptor interfacial area over time.^125–127^ P3HT was brominated, followed by Pd-catalysed cross-coupling with a nitroxide-bearing moiety that is capable of initiating a radical polymerisation (nitroxide-mediated radical polymerisation (NMRP)) with styrene (ST) and 4-chloromethylstyrene (CMS). The polymerised CMS could then be further functionalised with fullerenes *via* atom transfer radical addition (ATRA) to give P65 (Fig. 27).^125^ The photoluminescence in solution was completely quenched upon introduction of the fullerene, which indicates the donor–acceptor characteristics of the graft polymer. In a subsequent work, it was shown that it is also feasible to substitute the chlorides in the polymerised CMS units by azides and carry out a cycloaddition reaction of the azides and fullerenes/PCBM.^126^ The grafting density was carefully controlled between 1 and 30%. Investigations of the film microstructure demonstrated that the graft copolymers form a network with very small feature sizes (∼5 nm). It was shown that the domain sizes were decreased in comparison to blends of P3HT and PCBM. Due to the covalent link between the fullerene and the graft polymer, no diffusion and aggregation of fullerene molecules was observed. Even after extensive annealing no changes in domain sizes occurred. However, BHJ-PSC devices fabricated using the copolymer exhibited poor photovoltaic activities, which was attributed to less crystalline P3HT domains and the lower content (wt%) of active materials (P3HT and PCBM) in the active layer compared to conventional blends. In the graft-polymer, the active materials are diluted due to the presence of 36–60 wt% inactive side chains (poly(CMS-*stat*-ST)).

In the examples discussed thus far, backbone halogenation post-polymerisation has been utilised. However, it is also possible to introduce the halogen handle in the monomer and utilise it in a post-polymerisation reaction. For example, Bunz and colleagues reported the post-polymerisation functionalisation of a hyperbranched conjugated polymer containing iodine groups that are available for Sonogashira coupling reactions with alkynes.^128^ This allowed them to introduce a multitude of functional groups onto the backbone. The emission spectrum depended on the introduced functional groups, which enabled the authors to obtain polymers with emission maximum wavelengths ranging from 510 nm to 602 nm in solution and a strong bathochromic shift in the solid state.

**Fig. 28:**
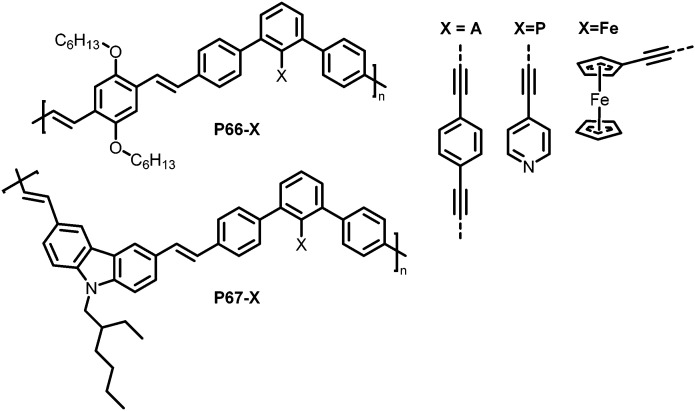
Polymers prepared *via* post-polymerisation functionalisation using Sonogashira coupling reactions.^129^

Functionalisation of linear conjugated polymers containing iodo groups has also been reported,^129^ with polymers P66 and P67 (Fig. 28, X = I) containing *m*-phenylene units. The presence of the *m*-phenylene impedes the conjugation in the polymer chain, and the iodo handle offers the possibility for Pd-catalysed coupling reactions to further functionalise the polymer. Three different units were introduced by coupling reactions, yielding polymers with different characteristics depending on the units (Fig. 28, X = A, P, Fe). P66-A, a crosslinked polymer that was expected to exhibit pockets around the terphenyl unit was tested for the detection of volatile organic compounds such as 2,6-dinitrotoluene (DNT). It should be noted that the polymer was most likely not fully crosslinked, so unreacted alkyne groups can be assumed to be present. Thin films of P66-A showed a fast response to DNT vapour, with its fluorescence quenched by half in just 30 s. The authors also tested the polymers in regard to their photoluminescence (PL) response to metal ions, as potential environmental sensors. In particular, the influence of different metal ions on the PL of polymers P66-P and P67-P, both bearing pyridyl groups, was investigated. Depending on the frontier orbital match of the metal ion-binding sidechain and the polymer backbone, the PL in solution was either quenched or intensified.

**Fig. 29:**
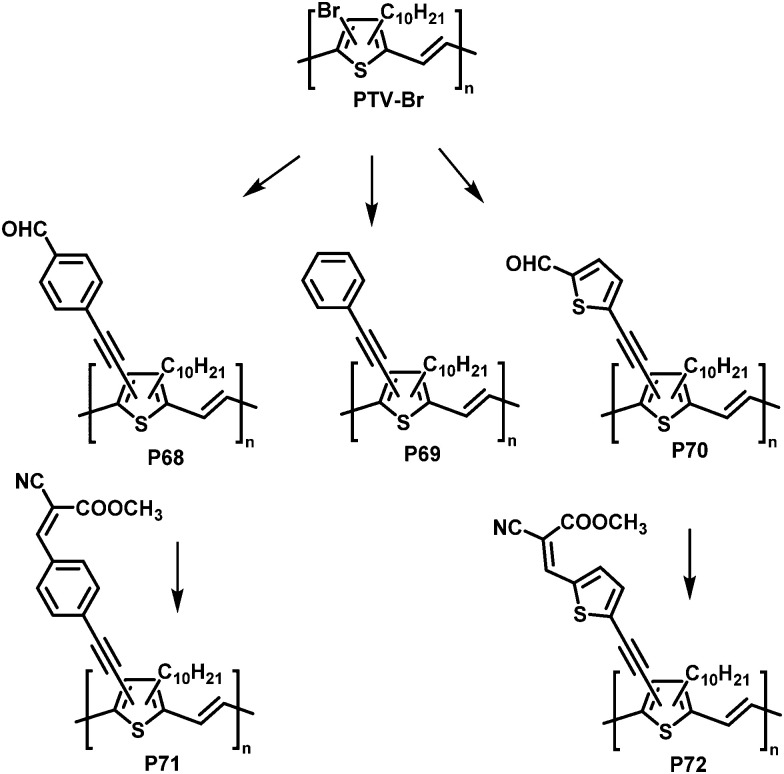
Post-polymerisation functionalisation of poly(thienylene vinylene)s (PTVs) with alkyne containing groups.^130^

**Fig. 30:**
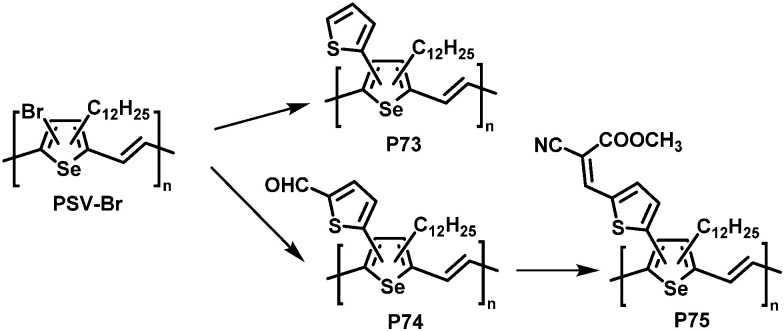
Poly(selenylene vinylene)s (PSVs) bearing thienyl moieties obtained by post-polymerisation functionalisation.^131^

Post-polymerisation functionalisation was also possible using brominated poly(thienylene vinylene)s (PTVs) and poly(selenylene vinylene)s (PSVs) for Stille and Sonogashira cross-coupling reactions (Fig. 29 and 30).^130,131^ Both, PTVs and PSVs, were prepared by acyclic diene metathesis (ADMET) polymerisation of brominated precursors. A variety of alkyne-containing groups were introduced quantitatively to PTVs by the cross-coupling reactions and further modification, allowing tuning of the optical gap and energy levels.^130^ A reduction in the gap of up to 0.3 eV was found in comparison to the parent PTV. Whilst OPV devices exhibited relatively modest performance (highest PCE ∼1% for P72) due to the amorphous nature of the polymers, the energy levels of the polymers could be tuned to the reduction potential of hydrogen, allowing photocatalytic hydrogen evolution. Blends of functionalised PTVs (P68–72) with graphitic carbon nitride (g-C_3_N_4_), exhibited better photocatalytic activity than g-C_3_N_4_ alone, but the pristine PTV exhibited the best performance. This was attributed to the energy misalignment between the LUMO level of the PTVs and the conduction band edge of g-C_3_N_4_, together with the reduced crystallinity in the functionalised polymers.

Similar results were reported for post-polymerisation-functionalised PSVs (Fig. 30).^131^ Thienyl derivatives were introduced to the backbone with a degree of functionalisation close to 100%, resulting in lower lying energy levels of polymers P73–75 due to inductive and conjugation effects of the substituents. However, due to the amorphous nature of P73–75 the performance in OPV devices was again low, with a maximum PCE of 0.74% for P75.

## Summary and conclusions

In this review, we have presented and discussed the different types of reactions and approaches for post-polymerisation functionalisation of conjugated polymer backbones. It is clear that the introduction of functional groups on the backbone by post-polymerisation functionalisation methods can have pronounced effects on the properties of conjugated polymers, which can be used to change, adapt, and optimise the properties for specific applications. Backbone functionalisation can also facilitate the preparation of both donor and acceptor materials for the use in optoelectronic devices or enable the preparation of graft co-polymers, which can improve the morphology and, hence, the performance of the devices. A broad range of applications and strategies on how to improve the device performance and morphology using post-polymerisation functionalisation approaches have been presented. However, trends in the nature of the changes of the properties upon functionalisation are not always clearcut, due to differences in the nature of each conjugated polymer and the competing effect of steric and electronic influences. For example, sulfur oxidation results in a consistent red shift in the optical gap when applied to the heteroatom of thiophene containing polymers, as the resulting thiophene-1,1-dioxide is more electron accepting and steric interactions with adjacent heterocycles are not significantly changed. However, when applied to thioether groups attached directly to the polymer backbone, as in the case for S-PPV (P31), the optical bandgap is widened, likely due to the steric effects of the larger sulfone group influencing backbone planarization and solid-state packing effects. Nevertheless, trends can be observed with a consistent series of conjugated polymers. For example, increasing displacement of fluoride from F8FBT (P40) with thioether groups results in a consistent decrease in intensity of the long-wavelength absorption band together with the growth of a new optical transition in the blue region of the spectra. This can be precisely controlled by the degree of reaction.

Cycloaddition as well as halogenation and subsequent Pd-catalysed cross-coupling are the most frequently used types of reactions for post-polymerisation functionalisation of conjugated polymers directly on the backbone. Such reactions often proceed rapidly with high selectivity, making them ideal for the functionalisation of polymers, where defects are obtained if side reactions occur. However, it is noted that in some of these cases a full degree of functionalisation could not be achieved. Although there are many applications where this is not problematic, good control over the degree of functionalisation is desirable from a reproducibility standpoint. Oxidation (or reduction) as well as nucleophilic aromatic substitutions are two types of reactions that allow for such good control of the degree of functionalisation, prompting the recent interest in these reactions.

Most of the presented reactions used 1–2 equivalents of reagent per reactive site, but for some reactions a larger excess of reagents was used to maximise the degree of functionalisation within the reaction time. This is particularly true for the presented cyclopentannulation (5 equiv.), Diels–Alder (5 equiv.) and S_N_Ar (up to 10 equiv.) reactions. It should be noted, however, that many of the reagents used for these reactions are low-cost, such as the alcohols and thiols used for the S_N_Ar reactions, which limits the impact on the cost of the reactions. Furthermore, post-polymerisation functionalisation can facilitate the direct introduction of reactive groups which would not survive the polymerisation conditions, removing the requirement for additional protection/deprotection steps or functional groups interconversions. For example, traditional approaches to the introduction of azide containing sidechains require the polymerisation of alkyl bromide containing polymers, followed by a subsequent interconversion to the azide.^132^ In contrast, post-polymerisation S_N_Ar reactions can directly introduce azide containing sidechains. Similarly, S_N_Ar has been shown to directly introduce highly moisture sensitive cross-linkable sidechains, such as trimethoxysilanes.^93^ These are extensively used as surface modifiers and cross-linkers in non-conjugated polymers but have barely been investigated in the world of conjugated polymers due to their lack of compatibility with typical polymerisation conditions.

There are many possible directions for future research in the field, but we believe it should focus where post-polymerisation functionalisation can particularly show its strengths, namely the introduction of functional groups or moieties that cannot easily be introduced *via* other strategies as well as the creation of sets of structurally related conjugated polymers – or a combination of both. For example, many approaches to tune the performance of conjugated polymers rely on the copolymerisation of appropriate monomeric materials. However, the establishment of structure–property relationships in such materials is often complicated by the need to synthesise batches of varying comonomer ratios. It is therefore difficult to exclude the influence of variations in the molecular weight, end-groups and dispersities from changes in comonomer ratio. Performing post-functionalisation chemistry on single batches of consistent weight and dispersity may help to overcome these issues. Furthermore, the development of post-polymerisation chemistries which can be utilised to selectively functionalise the surface of a polymer film or nanoparticle is an exciting direction. Such approaches could potentially tune the energy gradient within such systems, potentially facilitating exciton migration and harvesting, or allow the attachment of receptor moieties for sensor applications. These functionalisations may rely on reactions presented in this review, focussing on the development of new polymers that can be obtained with these reactions, but future research should also aim to identify new reactions suitable for post-polymerisation functionalisation. The many opportunities that still need to be explored and the wide range of potential applications – ranging from organic electronics to biological applications – make post-polymerisation functionalisation of conjugated polymer backbones a truly exciting field for further research.

## Conflicts of interest

There are no conflicts to declare.

## Supplementary Material
